# A review of the quality of current diabetes clinical practice guidelines

**DOI:** 10.26633/RPSP.2017.90

**Published:** 2017-06-19

**Authors:** Alberto Barcelo, Muzamil Jawed, Anthony Qiang, the PAHO Diabetes Guideline Project Group

**Affiliations:** 1 Department of Public Health Science University of Miami Miller School of Medicine MiamiFlorida United States of America Department of Public Health Science, University of Miami Miller School of Medicine, Miami, Florida, United States of America.; 2 Independent Consultant Cockeysville Maryland United States of America Independent Consultant, Cockeysville, Maryland, United States of America.; 3 McMaster University Hamilton Ontario Canada McMaster University, Hamilton, Ontario, Canada.; 4 Ailton Cezário Alves Júnior Diógenes Arjona Ailton Cezário Alves Júnior, Diógenes Arjona, Alberto Barcelo, Yamile Bello, Sarah Bryson, Juan Sebastian Castillo, Oscar Mauricio Cuevas Valdeleon, Guillermo Dieuzeide, Tomiris Lissette Estepan Herrera, Enrique Gil Bellorin, Claudia Godoy, José Roberto Gómez, Sonia Simone Gray, Yina Paola Hoyos Ospina, Jared Huffman, Carolina Larragaña, Natália Miranda Siniscalchi, Ario Mirian, Robin Mowson, Jorge Alberto Ramírez Díaz, Henry Perez Reyes, Myriam Rodríguez, Yeniceth Salazar, Wilson Sanchez, Johanna Segovia, Ricardo Quiroga Siles, Jairo Virviescas, and Naydene Williams.

**Keywords:** Diabetes mellitus, type 2 diabetes mellitus/standards, protocols, practice guidelines as topic, Americas, Caribbean region, Europe, Latin America, North America, Spain, United Kingdom, Diabetes mellitus, diabetes mellitus tipo 2/normas, protocolos, guías de práctica clínica como asunto, Américas, Región del Caribe, Europa (continente), América Latina, América del Norte, Reino Unido, Diabetes mellitus, diabetes mellitus tipo 2/normas, protocolos, guias de prática clínica como assunto, Américas, Região do Caribe, Europa (continente), América Latina, América do Norte, Reino Unido

## Abstract

**Objective:**

*To obtain an evaluation of current type 2 diabetes mellitus (T2DM) clinical practice guidelines*.

**Methods:**

*Relevant guidelines were identified through a systematic search of MEDLINE/PubMed. Pan American Health Organization (PAHO) country offices were also contacted to obtain national diabetes guidelines in use but not published/available online. Overall, 770 records were identified on MEDLINE/PubMed for citations published from 2008 to 2013. After an initial screening of these records, 146 were found to be guidelines related to diabetes. Inclusion and exclusion criteria were used to further refine the search and obtain a feasible number of guidelines for appraisal. Guideline evaluation was conducted by health professionals using the Appraisal of Guidelines for Research and Evaluation (AGREE) II instrument, which was developed to address the issue of variability in guideline quality and assesses the methodological rigor and transparency in which a guideline is developed. A total of 17 guidelines were selected and evaluated*.

**Results:**

*Ten guidelines scored ≥ 70% and seven guidelines scored ≥ 80%. The range was 21%–100%. The mean scores for Latin America and the Caribbean (LAC) country guidelines (*n *= 6) were compared to the mean scores for non-LAC country guidelines (*n *= 11). International guidelines consistently scored notably higher in all domains and overall quality than LAC guidelines*.

**Conclusions:**

*Based on this study’s findings, it is clear that T2DM clinical practice guideline development requires further improvements, particularly with regard to the involvement of stakeholders and editorial independence. This issue is most apparent for LAC country guidelines, as their quality requires major improvement in almost all aspects of the AGREE II criteria. Continued efforts should be made to generate and update high-quality guidelines to improve the management of increasingly prevalent noncommunicable diseases, such as T2DM*.

## RESUMEN

**Objetivo.:**

Evaluar las directrices de práctica clínica sobre la diabetes mellitus de tipo 2 que se utilizan en la actualidad.

**Métodos.:**

Se realizó una búsqueda sistemática en MEDLINE/PubMed con el fin de localizar las directrices pertinentes. Asimismo, se solicitó a las oficinas de la Organización Panamericana de la Salud (OPS) en los países que facilitaran las directrices nacionales sobre la diabetes utilizadas en cada país que no estuvieran accesibles ni publicadas en línea. Se obtuvieron 770 registros de trabajos publicados del 2008 al 2013 en MEDLINE/PubMed. Tras un tamizaje inicial, se localizaron 146 directrices relacionadas con la diabetes. Se aplicaron criterios de inclusión y exclusión para perfeccionar aún más la búsqueda y obtener un número viable de directrices para realizar la evaluación. La evaluación estuvo a cargo de profesionales de la salud, quienes utilizaron el instrumento AGREE II (Appraisal of Guidelines for Research and Evaluation), creado para abordar el problema de la variabilidad en cuanto a la calidad de las directrices, que evalúa el rigor metodológico y la transparencia del proceso de formulación. Se seleccionaron y evaluaron 17 directrices.

**Resultados.:**

Diez directrices recibieron una puntuación ≥ 70% y siete directrices, ≥ 80%. El margen de las puntuaciones asignadas fue de 21-100 %. Se comparó la media de las puntuaciones asignadas a las directrices provenientes de países de América Latina y el Caribe (n = 6) con la media de aquellas provenientes de otros países (n = 11). Las directrices internacionales recibieron una puntuación notablemente mayor que las de América Latina y el Caribe en todos los criterios evaluados y en la calidad general.

**Conclusiones.:**

Dados los resultados de este estudio, está claro que es preciso mejorar la formulación de directrices de práctica clínica sobre la diabetes mellitus de tipo 2, en particular con respecto a la participación de los interesados directos y la independencia editorial. Esta cuestión es sumamente evidente en las directrices de los países de América Latina y el Caribe, puesto que son necesarias mejoras considerables de la calidad en casi todos los aspectos de los criterios evaluados con el instrumento AGREE II. Es fundamental continuar con los esfuerzos destinados a formular directrices de excelente calidad y actualizarlas para mejorar el diagnóstico y el tratamiento de las enfermedades no transmisibles que son cada vez más prevalentes, como es el caso de la diabetes mellitus de tipo 2.

## RESUMO

**Objetivo.:**

Avaliar as diretrizes atuais para a prática clínica em casos de diabetes mellitus do tipo 2 (DMT2).

**Métodos.:**

Identificamos diretrizes relevantes por meio de uma pesquisa sistemática na base de dados MEDLINE/PubMed. As representações da Organização Pan-Americana da Saúde (OPAS) nos países também foram contatadas para que pudéssemos obter diretrizes para diabetes utilizadas nos países, mas não publicadas/disponíveis on-line. Ao todo, foram encontrados 770 resultados na MEDLINE/PubMed para citações publicadas entre 2008 e 2013. Depois de uma triagem inicial destes resultados, constatou-se que 146 eram diretrizes relacionadas ao diabetes. Utilizamos critérios de inclusão e exclusão para refinar ainda mais a pesquisa e obter um número viável de diretrizes a serem avaliadas. A avaliação das diretrizes foi feita por profissionais da saúde usando o instrumento AGREE II (Avaliação de Diretrizes para Pesquisa e Avaliação), desenvolvido para abordar a questão da variabilidade na qualidade de diretrizes e avaliar o rigor metodológico e a transparência no desenvolvimento de uma diretriz. No total, foram selecionadas e avaliadas17 diretrizes.

**Resultados.:**

Dez diretrizes tiveram pontuação ≥70%, e sete diretrizes tiveram pontuação ≥80%. A variação foi de 21% a 100%. As pontuações médias das diretrizes de países da América Latina e Caribe (ALC) (n=6) foram comparadas às de países não pertencentes a esta região (n=11). As diretrizes internacionais tiveram pontuações consistentemente mais altas em todos os domínios e uma qualidade global mais elevada que as diretrizes da ALC.

**Conclusões.:**

Com base nos resultados deste estudo, está claro que o desenvolvimento de diretrizes para a prática clínica em casos de DMT2 precisa ser aperfeiçoado, especialmente no que diz respeito à participação dos interessados diretos e à independência editorial. Este problema fica muito evidente no caso das diretrizes de países da ALC, cuja qualidade precisa melhorar muito em quase todos os aspectos dos critérios AGREE II. É preciso fazer esforços contínuos para desenvolver e atualizar diretrizes de alta qualidade a fim de melhorar a gestão de doenças não transmissíveis cada vez mais prevalentes, como o DMT2.

Diabetes mellitus is a group of diseases characterized by hyperglycemia due to defects in insulin secretion, insulin action, or both. Diabetes mellitus can lead to several poor health outcomes, including cardiovascular disease, nerve damage, blindness, amputations, and kidney failure. There are significant challenges in the control and prevention of diabetes, with worldwide prevalence among adults (20–79 years old) estimated at 415 million in 2015 (1). There are three main forms of diabetes mellitus: type 1, type 2, and gestational diabetes. Among them, type 2 diabetes mellitus (T2DM) is the most common form, accounting for more than 90% of all cases (2). T2DM, characterized by insulin resistance and relative insulin deficiency, was once primarily observed in economically affluent countries. Recent epidemiological trends, however, suggest an increasing prevalence of T2DM in low-and middle-income countries (1). Consequently, the health systems of countries such as those in Latin America and the Caribbean (LAC) face major challenges in coping with the rising burden of T2DM (3, 4). With the prevalence expected to continue to rise in LAC countries, there is an urgent need to improve the management and care of T2DM based on the best available clinical evidence (1, 4).

## CLINICAL PRACTICE GUIDELINES

Evidence-based clinical practice guidelines (CPGs) are documents designed to guide decision-making with regard to the diagnosis, management, and treatment of various disorders. CPGs are based on a systematic review of evidence and an assessment of the benefits and harms of various care options. Thus, these guidelines bridge the gap between research findings and practice. The quality of CPGs is therefore essential as they can influence health care delivery methods and, ultimately, affect patient outcomes. High-quality guidelines promote the use of effective clinical services, reduce the amount of practice variation, minimize the use of ineffective or questionable procedures and services, and increase the use of underused effective services (5). Nevertheless, a number of studies have reported that the quality of CPGs in a variety of clinical areas is modest or variable (6, 7). Consequently, the evaluation of CPGs is of great importance. The objective of this study was to obtain an evaluation of current T2DM clinical practice guidelines.

## MATERIAL AND METHODS

### Identification of guidelines

Relevant guidelines were identified through a systematic search of MEDLINE/PubMed. Pan American Health Organization (PAHO) country offices were also contacted to obtain national diabetes guidelines in use but not published/available online. The online search used the following strategy: *diabetes mellitus* as a MeSH term (mh) or all variations of *Diabet* after the “t” in the article title (ti) and *guideline, practice guideline,* or *consensus development conference* as a publication type (pt) or *health planning* or *clinical protocols* as a MeSH term (mh) or all variations of *guideline* after the “n” in the article title (ti). Through the specified search strategy, 770 records were identified on MEDLINE/PubMed for citations published from 2008 to 2013. After an initial screening of these records, 146 were found to be guidelines related to diabetes. Inclusion and exclusion criteria were used to further refine the search and obtain a feasible number of guidelines for appraisal. Inclusion criteria for selected guidelines included 1) focus on adult populations only; 2) published between 2008 and 2013; 3) written in English, French, Portuguese, or Spanish; and 4) focus on the screening, diagnosis and/or management of T2DM only. Exclusion criteria for this search included guidelines that focused on specific diabetic/nondiabetic complications (e.g., diabetic foot, hypertension, and stroke). Guidelines that focused on recommendations for specific therapies or interventions were also excluded from the search. A total of 17 guidelines (8–24) were selected and evaluated (Table 1).

### Appraisal instrument

Guideline evaluation was conducted using the Appraisal of Guidelines for Research and Evaluation (AGREE) II instrument, which was developed to address the issue of variability in guideline quality and assesses the methodological rigor and transparency in which a guideline is developed. The AGREE II instrument is rapidly becoming the gold standard for the evaluation of CPG quality and is endorsed by the World Health Organization (WHO); the Council of Europe (Strasbourg, France); and the Guidelines International Network (Pitlochry, Perthshire, Scotland) (25). The AGREE instrument consists of 23 key items divided into six domains: 1) scope and purpose (Items 1–3); 2) stakeholder involvement (Items 4–6); 3) rigor of development (Items 7–14); 4) clarity of presentation (Items 15–17); 5) applicability (Items 18–21); and 6) editorial independence (Items 22–23). The AGREE II instrument uses a seven-point rating scale for each of the 23 items. A score of 1 is given to an item when there is no/poor information provided, and a score of 7 is given when there is exceptional quality of reporting for the item (26). The final component of the AGREE II evaluation consists of a rating for the overall quality of the guideline based on a scale of 1 (lowest quality) to 7 (highest quality), and a recommendation of “Yes,” “Yes, with modifications,” or “No” in regard to the use of the evaluated guideline in practice. The score for each domain is then standardized and expressed on a scale of 0–100. For the purposes of this report, a minimum standardized score of 70% (roughly equating to a given score of 4 on a scale of 1 to 7) is considered acceptable quality. A standardized score of 80% or higher is considered excellent (roughly equating to a given score of 5–6 on a scale of 7). The Appendix provides a complete breakdown of the AGREE II domains and the 23 corresponding items, and an example of how domain scores were calculated and standardized.

### Evaluator selection

Guideline evaluators were selected on a volunteer basis. An official request was sent to selected individuals at PAHO regional offices and professionals in the field to secure volunteers willing to evaluate the quality of selected guidelines using the AGREE II instrument. PAHO country offices were also asked to provide staff members for evaluation, and individual experts in diabetes and/or guideline evaluations were contacted to provide support for the project. Before the assessment, the evaluators were required to review the provided training material for the AGREE II instrument, which included the full user guide/manual for the AGREE II instrument and a short tutorial video on how to use and apply the AGREE II instrument for assessing practice guidelines. The number of evaluators for each guideline varied, but all guidelines were required to be evaluated by the AGREE II–stipulated minimum of two individuals. Once the selected diabetes CPGs were evaluated, reviewers measured their quality, according to scores from AGREE II, and provided any suggestions for improving them.

**TABLE 1.** Country/region, author, title, and abbreviated name for all documents included in an AGREE II^a^ quality evaluation of Latin America and the Caribbean (LAC) and international type 2 diabetes mellitus clinical practice guidelines (*n* = 17), 2008–2013

Country/region	Organization (author)	Guideline title (reference)	Abbreviated name
Argentina	Ministry of Health	Guía práctica clínica nacional sobre prevención, diagnóstico y tratamiento de la diabetes mellitus tipo 2 (8)	“ARG”
Australia	Royal Australian College of General Practitioners and Diabetes Australia	General practice management of type 2 diabetes (2014–15) (9)	“AUS”
Brazil	Ministry of Health	Cadernos de atenção básica: diabetes mellitus (10)	“BRA”
Canada	Canadian Task Force on Preventive Health Care et al.	Recommendations on screening for type 2 diabetes in adults (11)	“CTFPHC”
Canadian Diabetes Association Clinical Practice Guidelines Expert Committee et al.	Canadian Diabetes Association 2013 clinical practice guidelines for the prevention and management of diabetes in Canada (12)	“CDA”
Chile	Ministry of Health	Guía clínica de diabetes tipo 2 (13)	“CHIL”
Europe	Paulweber B et al.	A European evidence-based guideline for the prevention of type 2 diabetes (14)	“EUR”
International	International Diabetes Federation Clinical Guidelines Task Force	Global guideline for type 2 diabetes (15)	“IDF”
Asociación Latinoamericana de Diabetes	Guías ALAD sobre el diagnóstico, control y tratamiento de la diabetes mellitus tipo 2 con medicina basada en evidencia (16)	“ALAD”
Mexico	Gil-Velázquez LE et al.	Guía de práctica clínica: diagnóstico y tratamiento de la diabetes mellitus tipo 2 (17)	“MEX”
Nicaragua	Ministry of Health	Protocol de atención de la diabetes mellitus (18)	“NICA”
Panama	Pan American Health Organization	Guía para la atención integral de las personas con diabetes mellitus (19)	“PAN”
Spain	Ministry of Health and Consumer Affairs	Guía de práctica clínica sobre diabetes tipo 2 (20)	“SPA”
United Kingdom	National Institute for Health and Care Excellence	Type 2 diabetes: the management of type 2 diabetes (21)	“UK”
United States	U.S. Preventive Services Task Force	Diabetes mellitus (type 2) in adults: screening (22)	“USPSTF”
Riethof M et al.	Diagnosis and management of type 2 diabetes mellitus in adults (23)	“ICSI”
American Diabetes Association	Clinical practice recommendations (24)	“ADA”

a Appraisal of Guidelines for Research and Evaluation instrument (AGREE Research Trust, London).

## RESULTS

A summary of the six assessment domains and corresponding criteria for the AGREE II quality assessment instrument is provided below. A summary of the scores for each guideline, by domain, can be found in Table 2.

### AGREE II evaluation instrument domains/criteria

#### Domain 1: Scope and purpose.

Domain 1 involves the degree to which the overall objectives, health questions covered, and guideline target population are specifically described. The majority of the guidelines scored very high for this domain, with only two guidelines scoring < 50%. In total, 15 (88%) of the guidelines scored ≥ 70% and eight (47%) guidelines scored ≥ 90%. Overall, the mean score for the guidelines was relatively high at 84% (range: 31%–100%). The median score for this domain was 89%.

#### Domain 2: Stakeholder involvement.

This domain focuses on the participation of relevant groups in the guideline’s development, the extent to which the views of the target population were considered, and how clearly the target group was defined. This was the most poorly scored domain. Only five guidelines scored ≥ 70%, and only two scored ≥ 80%. No guideline received a score of 100%. Five guidelines (29%) scored < 50%. The mean score for all guidelines was 61%—the lowest average score across all six domains. The median score was 58%, the lowest median score for all guidelines. The range was 33%–94%.

#### Domain 3: Rigor of development.

This domain involves the process used to gather and synthesize evidence and the methods used to formulate guideline recommendations. For rigor of development, nine guidelines scored ≥ 70%, and six scored ≥ 80%. Two guidelines scored < 50%. Overall, the mean score was 70% (range: 23%–100%), with a median score of 71%.

#### Domain 4: Clarity of presentation.

This domain deals with the level of clarity and ease of identifying recommendations in the guideline, along with whether alternative options for management of the health condition are present. This was the highest-scored domain of all six domains in terms of both the mean and median scores. A total of 15 guidelines scored ≥ 80% and 11 (79%) guidelines scored ≥ 90%. Only one guideline scored < 70%. Overall, the mean score was 88% and the median score was 92%. The range was 28%–100%.

#### Domain 5: Applicability.

This domain addresses the feasibility of guideline implementation, as well as strategies for and resource costs associated with guideline implementation. A total of 10 guidelines scored ≥ 70% and five scored ≥ 80%. No guideline received a score of 100%, and three guidelines scored < 30%. Overall, the mean score for this domain was 67% (range: 17%–94%), with a median score of 77%.

#### Domain 6: Editorial independence.

This domain concerns potential conflicts of interest associated with the development and content of the guideline and the extent to which this has been addressed. Along with stakeholder involvement, editorial independence scored very poorly relative to the other four domains. Five guidelines scored ≤ 50% and five guidelines scored ≥ 80%. Overall, the mean and median scores for the guidelines were 62% and 63%, respectively, with a range of 19%–100%.

**TABLE 2.** Summary table: AGREE II^a^ standardized mean domain and overall scores from an evaluation of Latin America and the Caribbean (LAC) and international type 2 diabetes mellitus clinical practice guidelines (*n* = 17), 2008–2013

Guideline (abbreviated name)	AGREE II domain						Overall guideline assessment	
	1	2	3	4	5	6				
	Scope and purpose	Stakeholder involvement	Rigor of development	Clarity of presentation	Applicability	Editorial independence	Overall score	Recommended for use in practice
ARG	94	58	56	89	79	40	63	“Yes” “Yes, with modifications”
AUS	92	83	60	97	79	63	83	“Yes” “Yes, with modifications”
BRA	31	42	40	83	29	25	50	“Yes, with modifications” “No”
CTFPHC	89	33	71	94	83	58	75	“Yes”
CDA	92	94	89	92	67	92	83	“Yes” “Yes, with modifications”
CHIL	89	56	68	89	81	63	83	“Yes”
EUR	92	75	82	92	90	71	83	“Yes”
IDF	53	39	79	94	77	54	67	“Yes, with modifications”
ALAD	100	78	100	100	65	67	83	“Yes”
MEX	78	47	63	94	27	19	67	“Yes” “Yes, with modifications”
NICA	88	42	23	28	17	21	21	“Yes, with modifications” “No”
PAN	78	64	52	89	81	50	58	“Yes, with modifications”
SPA	80	58	71	76	51	57	70	“Yes” “Yes, with modifications”
UK	92	64	91	97	77	83	100	“Yes” “Yes, with modifications”
USPSTF	100	56	67	94	65	100	67	“Yes, with modifications”
ICSI	100	78	91	92	94	100	92	“Yes” “Yes, with modifications”
ADA	78	67	94	100	77	88	75	“Yes” “Yes, with modifications”

a Appraisal of Guidelines for Research and Evaluation instrument (AGREE Research Trust, London).

### Guideline assessment and recommendations

Ten guidelines scored ≥ 70%, and seven scored ≥ 80%. Only the NICE^5^ guideline (“UK”) obtained a perfect score of 100%. Two LAC guidelines had an overall score of ≥ 50%. The Nicaraguan Ministry of Health guideline (“NICA”) scored the lowest (21%). The range was 21%–100%.

Four guidelines (“ALAD,” “CHIL,” “CTFPHC,” and “EUR”^6^) received a unanimous recommendation of “Yes” for use in practice. Eight guidelines received recommendations of both “Yes” and “Yes, with modifications” for use in practice, and three guidelines received rec ommendations of only “Yes, with modifications.” Only two guidelines (“BRA”^7^ and “NICA”) received evaluator recommendations of both “Yes, with modifications” and “No” for their use in practice.

### LAC versus international guidelines scores

**FIGURE 1.**
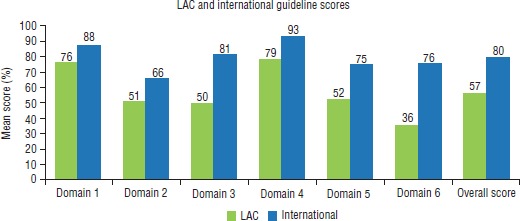
AGREE II^a^ mean scores (%) by domain (1–6^b^) from an evaluation of Latin America and the Caribbean (LAC) and international type 2 diabetes mellitus clinical practice guidelines, 2008–2013

^a^ Appraisal of Guidelines for Research and Evaluation instrument (AGREE Research Trust, London).

^b^ 1) Scope and purpose; 2) Stakeholder involvement; 3) Rigor of development; 4) Clarity of presentation; 5) Applicability; and 6) Editorial independence.

The mean scores (by domain) for LAC country guidelines (*n* = 6) were compared to those for the non-LAC country guidelines (*n* = 11) (referred to here as “international guidelines”) and are shown in Figure 1.

International guidelines consistently scored notably higher in all domains and overall quality than LAC guidelines. The smallest difference in scores was for “Scope and purpose,” where mean scores for LAC and international guidelines were 76% and 88% respectively. The biggest difference in scores was for “Editorial independence,” where mean scores were 76% for international and 36% for LAC. Interestingly, although international guidelines scored poorly for “Stakeholder involvement” (66%), the mean score for “Editorial independence” was a much more respectable 76%. Overall, excluding “Stakeholder involvement,” the mean domain scores for international guidelines all were ≥ 70%. LAC guidelines scores were much lower, with four mean domain scores ≥ 60%. The highest mean domain score for a LAC guideline was for “Clarity of presentation” (79%) and the lowest was for “Editorial independence” (36%).

## DISCUSSION

Domain scores were generally highly variable within guidelines. For example, the U.S. Preventive Services Task Force guidelines (“USPSTF”) scored ≥ 90% in three domains and < 70% in three other domains. Scores were also variable between guidelines. For example, the narrowest score range was for “Scope and purpose” (31–100). Domains corresponding to “Stakeholder involvement” and “Editorial independence” had the lowest median and mean scores for all guidelines. These results were consistent with similar studies evaluating CPG quality (5, 27). The poor scores for “Editorial independence” may stem from a lack of appreciation by guideline developers for the importance of conflict of interest disclosures, or could be indicative of actual conflicts of interests between sources of funding and guideline developers. Indeed, there is considerable evidence that financial conflicts of interest are highly prevalent among CPGs in a variety of clinical areas (28, 29). Furthermore, there is emerging evidence that conflicts of interest may affect guideline recommendations (30). Conversely, “Clarity and presentation” and “Scope and purpose” were the highest scored domains, consistent with other guideline evaluation studies (27, 31). This might reflect greater priority placed on these domains by guideline developers, or greater ease in achieving high scores for these domains.

Increased attention was given to scores for Domain 3 (“Rigor of development”), which may be considered a strong indicator of guideline quality (5, 32). Overall, the mean score for this domain for all guidelines was 70%, with a median score of 71%. However, like all of the other domains, there was a large discrepancy between Domain 3 scores for LAC and those for international guidelines. LAC guidelines performed much poorer (mean score of 50%) than international guidelines (mean score of 81%).

Overall, the discrepancy between LAC and international guideline scores was striking. Of the 17 guidelines evaluated, however, only two (from the latter group—“EUR” and “ICSI”^8^) scored ≥ 70% in all six domains. The LAC guidelines displayed some of the lowest sets of domain scores. The “BRA” and “NICA” guidelines had only one domain each—“Clarity of presentation” and “Scope and purpose” respectively—that scored ≥ 50%. Among all mean domain scores for LAC guidelines, the highest was 79% (for “Clarity of presentation”). Thus, it appears LAC guideline development is severely lacking in virtually all respects. This is especially concerning given the staggering prevalence and burden of T2DM in LAC countries (1, 3).

### Limitations

There were a number of limitations to this study. First, reviewers did not compare their individual scores with one another for each item, as suggested in the literature to reduce bias (5). The lack of comparison may be problematic, especially given the wide variation in this study in reviewers’ scores for a number of domains/items for the same guideline. Cross-checking scores between reviewers to identify large discrepancies and achieving consensus on those differences could have helped reduce the variation in scoring. This was impossible, however, as the reviewers were located in different countries with major connectivity limitations. However, all participating evaluators were given the same training and terms of reference in an effort to decrease individual bias. Another limitation of this study was that the search did not include databases other than MEDLINE/PubMed, and this may have limited the number of identified guidelines. However, the authors believe that 1) the number of identified international guidelines was sufficient for a good-quality evaluation, and 2) the lack of searches in Latin American databases was likely mitigated by the request to PAHO offices in the region for available guidelines that fulfilled the selection criteria, and that all available, qualifying guidelines from PAHO member states were obtained. Other limitations stemmed from issues related to the AGREE II tool itself. For example, no threshold is provided in the AGREE II instrument to distinguish high-quality guidelines from low-quality ones based on the scores. In this report, minimum values of 70% and 80% were arbitrarily chosen as cutoff points for “acceptable” and “excellent” quality respectively. Therefore, the quality ratings provided here are subjective and must be considered within that context. The statistical reliability and significance of the data is also questionable due to the limited number of appraisers scoring the guidelines. Thus, conclusions based on these data must be made with caution. Owing to the small sample size of evaluated guidelines, conclusions about the comparison between LAC and international guidelines must also be made with caution. Finally, the international guidelines were largely from more highly developed countries, such as Canada, the United States, and Australia, which may have skewed the data showing higher international guidelines scores.

## Conclusions

In recent years, there has been a trend of an increasing number of CPGs being developed by various organizations and other entities worldwide. These guidelines are designed to aid decision-making and reduce clinical practice variability and thus play a pivotal role in health care. It is therefore important that guideline development adheres to strict standards to ensure both consistency and high quality. Based on this study’s findings, it is clear that guideline development requires further improvements, particularly with regard to the involvement of stakeholders and editorial independence. This issue is most apparent for LAC country guidelines, as their quality requires major improvement in almost all aspects of the AGREE II criteria. Therefore, continued efforts should be made to generate and update high-quality guidelines in order to improve the quality of management for T2DM and other increasingly prevalent noncommunicable diseases.

## Disclaimer.

Authors hold sole responsibility for the views expressed in the manuscript, which may not necessarily reflect the opinion or policy of the *RPSP/PAJPH* or the Pan American Health Organization (PAHO).

## Footnotes

National Institute for Health and Care Excellence (London) (21).

Latin American Diabetes Association (16), Chilean Ministry of Health (13), Canadian Task Force on Preventive Health Care (11), and European IMAGE project (14) respectively.

Brazilian Ministry of Health (10).

The U.S. Institute for Clinical Systems Improvement guideline (23).
